# Barriers and facilitators of implementation of new antibacterial technologies in patient care: an interview study with orthopedic healthcare professionals at a university hospital

**DOI:** 10.1186/s12913-024-10878-4

**Published:** 2024-04-09

**Authors:** Lieve Vonken, Gert-Jan de Bruijn, Annika Noordink, Stef Kremers, Francine Schneider

**Affiliations:** 1 Department of Health Promotion, Research School CAPHRI, Maastricht University, P. Debyelaan 1, 6229 HA Maastricht, The Netherlands; 2 Department of Communication Studies, University of Antwerp, Stadscampus, S.M.481 Sint-Jacobsstraat 2, 2000 Antwerpen, Belgium

**Keywords:** Bacterial infections, Antimicrobial resistance, Antibacterial technology, Biomedical technology, Implementation, Quality improvement, Qualitative

## Abstract

**Background:**

Antimicrobial resistance is a major global health threat. Therefore, promising new antibacterial technologies that could minimize our dependence on antibiotics should be widely adopted. This study aims to identify the barriers and facilitators of the adoption of new antibacterial technologies in hospital patient care.

**Methods:**

Semi-structured interviews, based on the Consolidated Framework for Implementation Research, were conducted with healthcare professionals related to the orthopedics department of an academic hospital in The Netherlands.

**Results:**

In total, 11 healthcare professionals were interviewed. Scientific evidence for the effectiveness of the technology was the most explicitly mentioned facilitator of adoption, but other (often contextual) factors were also considered to be important. At the level of the inner and outer setting, high costs and lacking coverage, competition from other firms, and problems with ordering and availability were the most explicit perceived barriers to adoption. Participants did not collectively feel the need for new antibacterial technologies.

**Conclusions:**

Barriers and facilitators of the adoption of new antibacterial technologies were identified related to the technology, the hospital, and external factors. The implementation climate might have an indirect influence on adoption. New antibacterial technologies that are scientifically proven effective, affordable, and easily obtainable will most likely be adopted.

**Supplementary Information:**

The online version contains supplementary material available at 10.1186/s12913-024-10878-4.

## Background

The discovery of antibiotics prompted a revolution in treating bacterial infections and thereby medicine in general. Global dependence on antibiotics is currently high and increasing due to the high risk of infection in, for example, complex surgeries, treatment of chronic diseases, or chemotherapy [1]. However, the (inappropriate) use of antibiotics is rapidly fueling antimicrobial resistance (AMR) [1]. As bacteria become resistant to antibiotics, methods for preventing and treating infections become scarce since no simple alternatives to antibiotics exist [2]. This results in increased infection-related mortality and morbidity and will cause major losses in GDP (± 1%) and global trade [3]. The WHO, therefore, considers AMR to be one of the greatest threats to global health, food security, and development today [4]. 

In healthcare, AMR will challenge many procedures that rely on antibiotics for infection prevention such as surgeries and chemotherapy [3]. AMR is a major point of concern in orthopedics as the highly invasive surgeries and the use of prostheses result in high infection risk [5]. 

Infections strongly impact mortality and quality of life post-surgery. Specifically for prostheses, post-surgical infection at the implanted material surface is often considered the primary cause of implant failure [6]. For example, a Swedish study found that the mortality of total hip arthroplasty patients at 10 years was 14% higher in patients with prosthetic joint infections (48% versus 34%) [7]. To cope with the high infection risk, the need for antibiotics in orthopedics is especially high. With the rise of AMR, the burden of infections within orthopedics is expected to increase even further [5]. 

The imminent consequences of AMR stress the need for new antibacterial technologies that can help minimize our dependence on antibiotics. In recent years, promising technologies have been developed. These may be based on, for example, physicochemical methods and enable a reduction in the use of traditional antibiotics [2]. It is believed that the use of non-traditional antibacterial methods will prevent bacteria from obtaining resistance against these technologies [2]. Some examples of new antibacterial technologies are the coating of prostheses (with e.g., nanoparticles) and vaccination [2]. Many more technologies are now in the pipeline, most of which require different application methods than traditional antibiotics (e.g., the use of modified prostheses or the application of heat or ultrasound) [2]. 

To exploit the full potential of new antibacterial technologies they should be widely adopted in patient care without delay. However, we know from translational research that the widespread implementation of new technologies into clinical practice can take a long time [8, 9]. The first major time lag in the implementation process takes place in the development of a product that can be used in the clinic [8]. Primarily, the duration of the introduction of new technologies in orthopedics is delayed by the high number of studies needed before a technology can be proven effective [10]. The second major lag is between the development of a clinically viable product and the implementation of this product in clinical practice [8]. This stage can be divided further, into the adoption and the implementation stage [11]. Adoption refers to the initiation of a new process and therefore is a prerequisite for implementation in every hospital.

Research shows that even effective technologies substantiated by evidence will not automatically be adopted in practice [12]. Additional barriers and facilitators of adoption have been identified for several medical technologies and include characteristics of the technology, the individuals involved, and the context in and around the hospital [9, 13]. For technologies aimed at reducing antibiotic use, it can be argued that realizing changes is especially difficult since antibiotic use is embedded in structures (i.e., economic and political priorities), networks (i.e., communications), and practices (i.e., individuals) [14]. Knowledge about barriers and facilitators of adoption can be used to prepare and optimize the implementation process. However, these barriers and facilitators of adoption are not known for new antibacterial technologies.

The Consolidated Framework for Implementation Research (CFIR) provides a theoretical framework of potential barriers and facilitators of adoption and implementation at the external, internal, and individual levels, and related to the technology and the implementation process [15]. CFIR has been used to understand implementation and receptiveness to change in healthcare [16, 17]. This study is theoretically based on CFIR and aims to provide insight into healthcare professionals’ perceptions about the adoption of new antibacterial technologies that do not apply traditional antibiotics. The research question is: What are the barriers and facilitators of the adoption of new antibacterial technologies in hospital patient care?

## Methods

### Design

From February to April 2022, semi-structured individual interviews were conducted with healthcare professionals associated with the orthopedics department of the Maastricht University Medical Centre (MUMC+) in the Netherlands. The MUMC + is a university teaching hospital specializing in orthopedic infections, where patients with infections are referred from the periphery and a lot of research takes place. The study was approved by the Maastricht University Faculty of Health, Medicine and Life Sciences Research Ethics Committee (FHML-REC/2022/014). This report follows the Consolidated Criteria for Reporting Qualitative Research (COREQ) [18]. 

### Participants and recruitment

Participants were identified through purposive and snowball sampling from January to April 2022. We purposefully started with a surgeon who plays a central role in local orthopedic infection prevention and control. After the first interview, all subsequent participants were invited based on snowball sampling through suggestions from other participants. At the end of each interview, participants were requested to suggest other individuals for participation in this study. The aim was to recruit a sample of key stakeholders who could all shed a different light on the research topic from a clinical perspective. After 11 interviews participants no longer suggested new and relevant participants. All participants were invited through an email containing information on the purpose of the study. After a positive response, an information document and an informed consent form were emailed.

### Procedure

The interviews took place face-to-face in the hospital or, upon participant request, online through Zoom (Maastricht University license). LV (MSc, PhD candidate, experience with interviews) conducted all interviews. At the start of each interview, the informed consent form was signed, and time was taken to get acquainted. All interviews were audio recorded and transcribed verbatim. Field notes were made to aid in transcribing if the recording was unclear. Participants did not receive transcripts or reports of findings.

### Topic list

Interviews were guided by a topic list. The topic list covered current infection-related practices and healthcare professionals’ attitudes towards AMR in general (the first part) and beliefs regarding barriers and facilitators of the adoption of new antibacterial technologies (the second part). Questions in the first part were inspired by two questionnaires on attitudes and beliefs about AMR [19, 20]. The second part was based on CFIR and the topic list available from the CFIR website: https://cfirguide.org/. The CFIR topic list was adapted to apply to the future implementation of a hypothetical product. First, the number of CFIR constructs was reduced to characteristics of the individual, the technology, the inner setting (i.e., the hospital), and the outer setting (i.e., outside of the hospital). Second, the questions were phrased more openly. To make the questions easier to answer, participants were asked to choose a procedure they often take part in and then, to imagine using a technological antimicrobial technology in this procedure that does not make use of traditional antibiotics. The theoretical basis of the topic list ensured that all relevant topics were discussed during the interview while the open-ended questions allowed for additional input from the participants. The topic list was piloted with two healthcare professionals to confirm this, resulting in minor changes in question phrasing. The topic list is available in the additional file (additional file 1).

### Analysis

A thematic analysis approach was taken, applying a hybrid design of inductive and deductive coding as described by Fereday and Muir-Cochrane [21]. A predefined coding tree was developed that followed the structure of the topic list and was based on the tools available on the CFIR website (see additional file 1). To test the reliability and finalize this predefined coding tree, LV and AN test-coded one interview. Then, the predefined coding tree was used by LV to code the remaining interviews. While coding, LV identified themes and developed the final coding tree by moving or adding (CFIR) constructs (see additional file 1). Following this, AN coded all interviews, including the one previously coded, using the final coding tree (weighed kappa 94.8%; unweighted kappa 94.7%). As the results presented below are based on the final coding tree, the data presented is consistent with the findings, and major as well as minor themes were described. Quotes with participant numbers were added to give body to the reoccurring themes.

## Results

### Characteristics of the sample

Of the 10 healthcare professionals who were invited to participate, 9 participated. One invited participant proposed 2 more junior participants upon invitation instead of participating themselves. Thus, in total, 11 healthcare professionals participated: 4 surgeons (in training), 2 ward employees (nurse and nurse practitioner), 2 operating room (OR) employees (management and executive), and 3 infection (prevention) experts. The participants had an average of 11.3 years of experience in their profession (range: 1–30 years). All but one participant had daily to weekly encounters with patients with bacterial infections or antibiotic prescriptions. The participant who did not have such daily to weekly encounters is indirectly involved in these procedures and has a coordinating role. Previous participants suggested all but the first participants because they would have a role in the implementation of new technologies. On average, interviews lasted 40 min (range: 30–55 min).

### Stakeholders involved in the adoption process

The most often mentioned relevant stakeholders were the surgeon, the department (chair), the purchasing department, the Board of Directors, OR planning and management, those responsible for sterility, OR material management, OR quality and safety officers, the infection prevention team, medical microbiologists, OR ICT and the microbiology lab. For all stakeholders, different barriers and facilitators would influence their attitude toward adopting new antibacterial technology.

*”I think the surgeons will definitely be positive about this. With them, it won’t revolve around money. Neither in the OR. Maybe if it takes more OR time, that’s a different story of course. But in the end, the costs will be the most sensitive topic for the Board of Directors.” [D8]*.

### Technology-related barriers and facilitators

All participants stressed the need for scientific evidence before considering adopting new antibacterial technology. For a technology past the development phase, positive results from strongly regulated studies performed in the clinic, independent of industry, could facilitate adoption. Yet, for technologies that are still in the developmental phase, evidence of animal studies or a clear working mechanism could suffice as a facilitator. Participants explained that technology can be tested in a research context. In addition to scientific evidence, some participants indicated that positive information from peers or experts and a trustworthy and convincing industry representative would facilitate adoption.

*“Hard evidence, of course, based on good studies. In a research center, you’re always at the beginning of the chain. So often you must proceed based on in-vitro data or material characteristics of which you suspect that they might have an effect.” [D1]*.

All participants agreed that the technology itself would have to be effective and thereby benefit the patient. Moreover, cost-effectiveness was considered a facilitator, and high expenses were considered hindering. Implementing a very expensive technology was described as difficult. In addition, participants said user-friendliness would facilitate adoption. Some participants further indicated that it would facilitate adoption if the technology had an understandable working mechanism. Only one participant explicitly mentioned that adoption would be facilitated if the technology fits within the current treatment. This notion regarded the facilitating effect on adoption if a coating could be applied to all current implants.

### Outer setting-related barriers and facilitators

Participants expected that the adoption of new antibacterial technology in a hospital would be influenced by (inter)national laws and regulations, peers, or patient associations. Explicitly mentioned barriers to adoption include the costs not being covered (e.g., developmental costs or reimbursement by health insurers), problems with ordering and availability of the technology, competition among firms, problems in collaborating with industry partners and legal rights to a technology, and lacking awareness among the general public and physicians. The technology being mentioned in national recommendations or guidelines was considered facilitating.

*“If it works, it should end up in a guideline. If it doesn’t end up in a guideline, it’ll be very difficult to use it.” [D4]*.

### Patient-related barriers and facilitators of adoption

In all interviews, participants were asked about the patient as a potential barrier or facilitator to adoption. However, the patient was generally not perceived to influence adoption at all. Instead, the participants said to consider patients’ needs in their own adoption preferences. Participants explained that paternalism is preferred over shared decision-making once the technology is beyond the experimental stage. This is because the relevant information is believed to be overly complicated for patients. Only in the case of a new or experimental product will patients’ opinions be asked. Overall, participants expected that the patient would not object to a new procedure, especially if the procedure is explained well. However, some participants believed that patients might not be ready for something other than antibiotics, which could potentially be a barrier to adoption.

*“Eventually they [patients] are referred to an expert center. And they expect expertise there. […] Deciding together is a hot topic, but you notice that patients are strongly influenced by what the expert suggests. […] Because it’s very specialized information.” [D2]*.

### Inner setting-related barriers and facilitators of adoption

Explicitly mentioned hospital-related barriers to adoption include high costs and current tenders and contracts with other suppliers. Less explicitly, momentary factors that influence the hospital’s priorities were mentioned as barriers to adoption (e.g., the implementation of care paths and one-stage revisions, COVID-19, and the consideration of environmental impact). Moreover, participants mentioned two factors that they felt could help facilitate the adoption process, though these are not prerequisites to adoption. First, current procedures for ordering and distributing materials and for measuring the resistance pattern in the lab would ideally fit with the new antibacterial technology. Second, education and training needed for use of the technology should be well-organized and should lead to new standard procedures. Some participants indicated that doctors should initiate this implementation process.

*“Over the years I’ve seen quite a few changes. And it only makes your job more interesting. Because imagine that everything would stay the same, that would be boring. That’s what makes it so nice in a university hospital, new innovations, and something new every time.” [D11]*.

### Implementation climate

While this was not explicitly mentioned as a barrier or facilitator, the implementation climate of the hospital might also influence adoption. Primarily, if participants would perceive the priority of new antibacterial technology to be high, this might facilitate adoption. The perceived priority can be construed from three factors. Firstly, ideas about whether AMR is currently a problem that should be acted upon were mixed. While one half of the participants described AMR as a current problem, the other half explained that AMR is not urgent because the Netherlands is doing very well with regards to levels of AMR, or because AMR is a future as opposed to a current problem.

*”When treating an infection, we do. But if you’re just placing a prosthesis […], then it [AMR] is not really considered.” [D7]*.

Secondly, these divided opinions also showed when discussing the need for alternative antibacterial technologies. One half of the participants expressed the belief that these are very necessary right now while the other half believed that they should only be developed for future purposes. One participant did not recognize a current need for such technologies at all. Thirdly, while prevention and sterility in the OR and phage therapy were mentioned as alternative methods to curb AMR, no alternative methods were mentioned that would be prioritized over a new antimicrobial technology not making use of antibiotics. Overall, participants’ ideas about the priority of the new antibacterial technology were mixed. Merely the latter factor might be considered a facilitator.

*”I always say: “Show me the money”. What does it do? How does it work? How effective is it? What’s the science behind it? What’s known about the working mechanism, about resistance, about effectiveness, about the duration of the effect? What’s known about the micro-organisms etcetera.” [D6]*.

In addition, another aspect of the implementation climate, innovation-readiness, might facilitate adoption in this hospital, even though that facilitating effect was not explicitly mentioned. The positive influence of innovation-readiness on adoption can also be construed from three factors. Firstly, half of the participants mentioned that the hospital in which they work is always looking for ways to improve their care. While some participants indicated that adjustments occur frequently and smoothly (e.g., reconstruction, training of staff), others indicated that making changes to standard practice is difficult.

*“What of course plays a very important role here is that this is a center of expertise for infections. That is one of the focal points. […] If you can accomplish improvement there, then that will definitely be high on the list of priorities.”[D8]*.

Secondly, all participants who indicated that they would have a role in adoption described themselves as initiators or early adopters of change and very open to new antibacterial technologies. Thirdly, all but one participant could mention new examples of such technologies and felt capable to work with them in the future. Coatings, bioactive glass, and phage therapy were mentioned most often.

*“I won’t be the very first, but maybe within the first 10–15%. If I stand for it being good.” [D9]*.

## Discussion

This study was aimed at providing insight into barriers and facilitators of adopting new antibacterial technologies in patient care as perceived by hospital healthcare professionals. We interviewed a variety of clinical healthcare professionals who would be involved in adopting such technologies in the field of orthopedics. Expected barriers and facilitators of adoption were based on characteristics of the technology, the inner hospital setting, and the outer setting. Moreover, participants shared personal perceptions related to the implementation climate of the hospital that might underlie the barriers and facilitators mentioned.

At the level of the technology, its usability and the availability of scientific evidence of its effectiveness were considered to facilitate successful adoption. The need for scientific evidence that proves effectiveness is supported by a recent systematic review into barriers to the diffusion of medical innovations in healthcare [9]. In this study, it is described that the lack of high-quality evidence can hamper adequate adoption decisions [9]. Especially in orthopedics, scientific evidence is valued highly. This is partly because well-known orthopedic failures in recent years have highlighted the need for good evidence [10]. With regard to usability, participants described that if technology would allow for measuring the resistance pattern this would be facilitating. User-friendliness was also deemed important while problems with ordering, availability, and distribution of the technology were listed as potential barriers. Interestingly, these more practical barriers and facilitators of adoption were not identified in the aforementioned systematic review [9]. Participants may have mentioned these practical factors as a result of the timing of our study. Since the technologies are not yet developed, the innovation was described without details. Participants thus may have listed barriers and facilitators that prove not to be applicable to most technologies.

An important hindering factor was mentioned at the contextual levels of the inner hospital setting and outer setting. Specifically, lacking financial arrangements were listed as a potential barrier to adoption. Many previous studies have also described the importance of appropriate financial structures [9]. Participants of this study additionally described that competing (currently used) technologies complicated financing the adoption of new antibacterial technologies. Furthermore, it would facilitate adoption if national recommendations or guidelines would be issued that include the technology, according to the participants. The importance of guidelines was also highlighted in previous research [22]. 

Previous studies have recognized the patient as influential in adoption [13, 22]. Specifically, characteristics of the patient and their disease might facilitate or hamper the application of new innovations, or the patient themselves might be an actor in the decision-making process [13, 22]. Though the clinicians in this study said to have the patients’ best interests at heart, they generally did not mention the patient or their opinions as a barrier or facilitator to adoption. The complicated nature of the infection treatment was listed as a reason for not asking for patient preferences. Not knowing the patients’ preferences meant these preferences would not influence adoption. This seems contradictory to other studies in which the patient’s role in the decision-making process was described as ‘very active’ [22]. However, ‘very active’ patients were also described as ‘well informed’ [22], something that is, according to our participants, difficult for orthopedic infections. Earlier research also found that in general, some physicians might not be willing to consider patient preferences [13]. 

Next to the explicitly mentioned barriers and facilitators of adoption, questions related to the CFIR implementation climate revealed more implicit factors that might influence adoption. One remarkable perception that was identified in this study relates to the perceived priority of the new antibacterial technology. Most interestingly, participants were divided about whether AMR is currently an urgent problem that demands action. Some participants explained their sense of urgency was low because AMR is a problem for the future or in other countries besides the Netherlands. The dividedness about the urgency of AMR is in agreement with a recent study among infection control specialists where only about half of the Dutch participants perceived the risk of AMR to be high [23]. Further, an explanation for the low urgency these healthcare professionals perceive is related to the conclusion of systematic reviews of studies about healthcare professionals’ knowledge and beliefs about AMR. These described that clinicians often perceive AMR to be a problem “not in my backyard” [24, 25]. In other words, the perceived severity of AMR may be high but their perceived susceptibility is low. This also applies to the participants in our study who, for example, described that AMR is not a problem in The Netherlands. We know from behavior change theories that if the perceived risk (i.e., perceived severity and/or susceptibility) is low, the tendency for acting against that risk is often low as well [26]. Therefore, increasing our participants’ perceived AMR urgency and priority of the new antibacterial technologies might be a prerequisite for their willingness to adopt them.

Another remarkable characteristic of the participants of this study was their innovation-readiness. Although this was not explicitly mentioned as a facilitator of adoption it might indirectly facilitate the adoption process. Participants described themselves as innovative and adopting new antibacterial technology was not met with hesitance. Furthermore, they described their academic hospital as a setting where change and innovation occur often. These results fit with the idea that innovation is one of the purposes of an academic hospital [27]. Following Roger’s Diffusion of Innovations Theory, such an innovative group of healthcare professionals could play an important role in the adoption of new antibacterial technologies by kickstarting the adoption process [28]. However, since innovation-readiness was not explicitly mentioned as a facilitator to adoption it is unclear whether this might indeed lead our participants to kickstart adoption. We cannot conclude that all participants will automatically be innovators. For example, it has been shown that a hospital’s academic status does not guarantee innovation [27]. 

### Strengths and limitations

The main strength of this study is the structured and theoretical approach based on CFIR, an established implementation framework [15]. This approach yielded an extensive list of potential barriers and facilitators of the adoption of new antibacterial technology which allowed us to ask for a broad spectrum of possible barriers and facilitators. A further strength is that we interviewed healthcare professionals in different professions who shed different lights on our topic, again resulting in a broad spectrum of possible barriers and facilitators.

A limitation of this study is that we could only describe a hypothetical product without details on the product level since the technologies of interest are currently still under development. This required stakeholders to imagine a product and its implementation. Some implementation problems might only become evident to participants once a product’s characteristics are described. Especially the technology-related barriers and facilitators are dependent on specific technology characteristics.

A further limitation is that, while the sample of key stakeholders was diverse, we only interviewed clinical healthcare professionals who were suggested by previous participants. Firstly, we did not verify the amount of decision power participants had. Secondly, stakeholders responsible for the barriers and facilitators identified (i.e., the Board of Directors, insurers, and guideline developers) might help to further understand the context influencing these barriers and facilitators. Thirdly, the diversity of the participants (e.g., in years of experience) and the small sample size did not allow for the extensive identification of differences between different types of healthcare professionals. Lastly, further research should explore the patient’s perspective.

### Recommendations

When implementing new technologies in practice, it is often recommended to focus on the development of a large evidence base for the effectiveness and safety of these technologies [10]. However, this study shows that although orthopedic healthcare professionals in an academic hospital perceive evidence as an important facilitator for the adoption of antibacterial technologies, other barriers and facilitators might also influence the adoption success. We recommend both researchers and technology developers study barriers and facilitators of the adoption of their technology early in the development process. At an earlier stage, adaptations to the technology or the implementation setting are often more feasible. For example, the technology could be designed with easy distribution and ordering in mind.

For future research, we recommend two types of studies. First, the association between adoption and the identified barriers and facilitators should be confirmed in further research. Specifically, quantitative studies describing the strength of the association should be performed. Second, when approaching the adoption of a specific technology, we recommend repeating the current study, though focusing more on the characteristics of the technology and interviewing more stakeholders (e.g., the Board of Directors). Academic specialized hospitals might be the starting point in such research as these centers are familiar with innovation and research and might therefore be more open to innovation. Additional studies are needed to be able to guide the implementation of antibacterial technologies in (peripheral) hospitals where innovation, participation in research, and new orthopedic technologies are less common. In these hospitals, other factors might influence the adoption of new antibacterial technology.

## Conclusions

In this study, in a sample of orthopedic healthcare professionals from an academic hospital, barriers and facilitators of the adoption of new antibacterial technologies were identified related to the technology, the hospital, and external factors. The results suggest that the adoption of new antibacterial technology in orthopedics is easiest if the technology is scientifically proven effective and if barriers related to the hospital and external factors can be overcome. However, the adoption of new antibacterial technologies in an academic-specialized hospital could also be influenced by the implementation climate. Specifically, a lack of perceived priority for new antibacterial technologies might hinder adoption while the innovation-readiness of the hospital and the staff might facilitate adoption. Adoption strategies should be tailored to the innovation-readiness of the hospital and the priority given to new antibacterial technologies. Developers and implementers of new antibacterial technologies for orthopedics should focus particularly on developing new antibacterial technologies that are scientifically proven effective, affordable, and easily obtainable.

### Electronic supplementary material

Below is the link to the electronic supplementary material.

Supplementary Material 1: Barriers and facilitators of imp_Additional file 1.docx– Topic list and coding tree

## Data Availability

The datasets generated and/or analyzed during the current study are not publicly available due to them containing information that could compromise research participant privacy but are available from the corresponding author in anonymized form on reasonable request.

## Abbreviations

AMR: Antimicrobial resistance
CFIR: Consolidated Framework for Implementation Research
MUMC+: Maastricht University Medical Centre
