# Supplementary material for: Barriers and facilitators of implementation of new antibacterial technologies in patient care: an interview study with orthopedic healthcare professionals at a university hospital
Source: BMC Health Serv Res. 2024 Apr 9;24:447. doi: 10.1186/s12913-024-10878-4 (PMC11005272; doi:10.1186/s12913-024-10878-4)
Supplement: Supplementary file 1 — Supplementary Material 1: Barriers and facilitators of imp_Additional file 1.docx– Topic list and coding tree [file 12913_2024_10878_MOESM1_ESM.docx]

**Topic list**

**A. General introduction**

**B. Personal/professional questions**

1. What is your current position?
2. How many years have you worked in this position?
3. How many years ago did you complete your last training for this position?
4. Do you have other tasks?
5. How often do you deal with bacterial infections and/or the prescription of antibiotics in your work?
   *Daily / Weekly / Monthly / Semi-annually / Less often*
6. Are there other things about your background that might be relevant to this research? For example, previous work experiences or other experiences that influence your opinion about antibacterial technology.

**C. Action against bacterial infections and new technologies**

- 1. What kind of operations or actions are you mainly involved in? [We focus on operations on the musculoskeletal system]
  2. To make this interview more specific, I would like to focus on one procedure. Is there a procedure that you perform often/are often involved in, in which infection prevention and treatment are important? Please describe this intervention.
  3. How often do you have to deal with this procedure in your work?

*Daily / Weekly / Monthly / Semi-annually / Less often*

**Keep this procedure in mind for all further questions.**

- 1. Within your chosen procedure, what is done regarding the prevention and treatment of bacterial infections?
     1. What is the extent of the role of antibiotics?
  2. Within your chosen procedure, is the prevention of antibiotic resistance considered? If so, how?
     1. Do you believe this should happen (more)?
  3. Do you believe antibiotic resistance is a problem with this procedure? Explain.

Currently, a lot of work is being done worldwide on new antibacterial technology; including technology that does not use antibiotics. This technology could become important when antibiotic resistance becomes more prevalent.

- 1. When I outline these developments, do any technologies come to mind that you are familiar with?

*Examples of these types of technologies are coatings, the application of specific shapes (morphological characteristics) to the surface of prostheses, heating of the prostheses, radiotherapy [prostheses]; calcium phosphate powders, granules, coatings [hard tissue]; new materials (supramolecular properties, bioactive glass) [soft tissue].*

- 1. When you hear these examples of technology, what do you think?

**E. CFIR factors**

In the future, this technology may be used in your hospital and work. I would like to discuss the factors with you that may affect how the technologies are implemented.

For the following questions, imagine that technology has been developed. It could be one of the examples I just mentioned, but it's important that you imagine something that you're not currently using or for which you are not involved in the development, for example. We've talked about [procedure] up until now. Can you imagine a new type of antibacterial technology that would be used in this procedure? Keep this technology in mind.

*Individual opinions about the technology*

1. Do you believe this technology is badly needed? Why or why not? For whom?
   1. Do you ideally see the technology as an addition to the current treatment or as a replacement for it? Explain.
   2. Is there another option you would rather use? Can you describe this and explain why?
2. What do you believe are the important features that the technology should have?
3. What kind of information would you need to make a final decision on whether you would like to use the technology and whether it would suit your workplace? Where would this information ideally come from?

*Outer setting factors*

1. What factors outside the hospital do you believe play a role in the adoption of this technology?
   1. Are there other factors at the national level?
   2. And on the local level?
2. To what extent should the needs and preferences of the patient be considered when deciding whether to use technology?
   1. How do you believe patients would respond to the technology?

*Inner setting factors*

1. If you look at your own workplace. What factors from within the hospital do you believe play a role in the adoption of these technologies?
2. Whom do you believe will influence the decision to implement the technology in the hospital?
   1. Whom do you believe will have an influence (positive and negative) on the implementation process?
3. Are there any practical features, processes, or actions in the hospital that would affect the adoption of the technology?
   1. Are there any high-priority initiatives in your workplace now or in the coming years that may impact the adoption of the technology? If so, which ones and in what way?
4. Are there norms and values within your workplace that would influence the introduction of the technology?
   1. Would you be motivated externally to implement this technology? If so, how?

*Individual opinions about the technology*

1. What would make you want to use this technology and what would hinder it?
2. After the implementation phase is completed, different people will have a role in using the technology. Do you believe you would have a part in this? How confident are you that others could perform these roles well? How confident are you that you could perform your role well? Why?
   1. Would you be able and willing to be an initiator in the introduction of the technology? Why?

**F. Additional factors or people**

1. We have now discussed many factors that may influence whether new antibacterial technology is implemented successfully. I will now briefly summarize these factors. In your opinion, are there any factors that are missing from this list? If so, which ones?
2. Is there anything else you would like to say about this topic?
3. In addition to this conversation with you, we have also conducted or planned other interviews. Do you believe there are people in this hospital we should talk to about this topic? If so, who?

**G. Closing**

**Table 1. Predefined coding tree.**

| *Construct* | | | | *Adjustments compared to CFIR* |
| --- | --- | --- | --- | --- |
| **IV. CHARACTERISTICS OF INDIVIDUALS** | | | |  |
| A | Job experience and characteristics | | | Additional construct |
|  | A1 | Job description | | Additional construct |
|  | A2 | Job preparation | | Additional construct |
|  | A3 | Influential experiences/characteristics | | Additional construct |
| B | Focus procedure | | | Additional construct |
|  | B1 | Description | | Additional construct |
|  | B2 | Infection action | | Additional construct |
|  | B3 | AMR action | | Additional construct |
| D | AMR urgency | | | Additional construct |
| E | Knowledge & Beliefs about the Intervention | | |  |
| F | Self-efficacy  Executing | | | Originally under Process |
|  | F1 | | Individual Stage of Change | Originally a main construct |
| G | Other Personal Attributes | | |  |
| **I. INNOVATION CHARACTERISTICS** | | | |  |
| A | Intervention source  Evidence Strength & Quality | | |  |
| B | Relative Advantage  Tension for Change  Relative priority | | | Originally under Inner Setting  Originally under Inner Setting |
|  | B1 | Adaptability | |  |
|  | B2 | Trialability | |  |
|  | B3 | Complexity | |  |
|  | B4 | Design Quality and Packaging | |  |
|  | B5 | Cost | |  |
| **II. OUTER SETTING** | | | |  |
| A | Patient Needs and Resources | | |  |
| B | Cosmopolitanism  Peer Pressure | | |  |
| C | External Policy and Incentives | | |  |
| **III. INNER SETTING** | | | |  |
| A | Structural Characteristics | | |  |
| B | Networks and Communications | | |  |
|  | B1 | Relevant stakeholders | | Additional construct |
| C | Culture | | |  |
| D | Implementation Climate  Organizational Incentives and Rewards  Goals and Feedback  Learning Climate | | | Originally sub-construct Implementation Climate  Originally sub-construct Implementation Climate  Originally sub-construct Implementation Climate |
|  | D1 | Compatibility  Available Resources | | Originally sub-construct Readiness for Implementation |

Most constructs were adopted from the Consolidated Framework for Implementation Research (CFIR; Damschroder et al., 2009). The following CFIR constructs were excluded from the coding tree. Process: Planning, Engaging (i.e., Opinion Leaders, Formally Appointed Internal Implementation Leaders, Champions, External Change Agents), Reflecting & Evaluating. Inner setting: Readiness for Implementation (i.e., Leadership Engagement, Access to Knowledge and Information). Individual characteristics: Individual Identification with Organization. NB. The subconstructs belonging to the main construct ‘process’ are included under other constructs.

AB: antibiotics; AMR: antimicrobial resistance.

**Table 2. Final coding tree.**

| *Construct* | | | | *Short Description* |
| --- | --- | --- | --- | --- |
| **I. CHARACTERISTICS OF INDIVIDUALS** | | | | |
| A | Job experience & Characteristics [ADDED] | | | Parent node – no coding. |
|  | A1 | | Job description [ADDED] | Current job, frequently performed actions, additional tasks, frequency of dealing with infections/AB. Not description focus procedure (see I.B). |
|  | A2 | | Job preparation [ADDED] | Years of experience, years since last education. |
|  | A3 | | Influential experiences/ characteristics [ADDED] | Relevant previous experiences. |
| B | Focus procedure [ADDED] | | | Parent node – no coding. |
|  | B1 | | Description [ADDED] | Description of the procedure that will be discussed in the interview, e.g., frequency of occurrence. |
|  | B2 | | Infection action [ADDED]  Networks and Communications [INNER SETTING] | Description of the actions against infections within the focus procedure, role of AB.  The nature and quality of webs of social networks and the nature and quality of formal and informal communications within an organization.  🡪 Only code here if related to infection action. |
|  | B3 | | AMR action [ADDED] | Description of the actions against AMR within the focus procedure. |
| C | AMR urgency [ADDED] | | | Is what is being done sufficient, urgency of AMR. |
| D | Knowledge and Beliefs about the Intervention  Other Personal Attributes | | | Individuals’ attitudes toward and value placed on the intervention as well as familiarity with facts, truths, and principles related to the intervention.  A broad construct to include other personal traits such as tolerance of ambiguity, intellectual ability, motivation, values, competence, capacity, and learning style.  🡪 Only if coding at another node (e.g., innovation characteristics) is insufficient. Code e.g., strong personal beliefs here.  🡪 Code pre-existing knowledge under D1. |
|  | D1 | Pre-existing knowledge [ADDED] | | About technological AMR innovations. |
|  | D2 | Self-efficacy  Executing [PROCESS] | | Individuals’ beliefs in their own capabilities to execute courses of action to achieve implementation goals.  Carrying out or accomplishing the implementation according to plan.  🡪 Answer to “Would you have a role in implementation/usage?” |
|  | D3 | Individual Stage of Change | | Characterization of the phase an individual is in, as he or she progresses toward skilled, enthusiastic, and sustained use of the intervention.  🡪 Answer to “Would you be willing to take the initiative or not?” |
| **II. INNOVATION CHARACTERISTICS** | | | | |
| A | Evidence Strength and Quality  Intervention source | | | Stakeholders’ perceptions of the quality and validity of evidence supporting the belief that the intervention will have desired outcomes.  Perception of key stakeholders about whether the intervention is externally or internally developed. |
| B | Relative Advantage | | | Stakeholders’ perception of the advantage of implementing the intervention versus an alternative solution.  🡪 Answer to “Is it needed, is it an addition or replacement, are there any alternatives you prefer?”  🡪 Prefer one of the subcodes below over coding at this code. |
|  | B1 | | Adaptability | The degree to which an intervention can be adapted, tailored, refined, or reinvented to meet local needs.  🡪 If it merely describes the current context (not the innovation), see III.B. |
|  | B2 | | Trialability | The ability to test the intervention on a small scale in the organization, and to be able to undo implementation if warranted.  🡪 Code statements on study possibilities and experimental/established  🡪 Only if a statement is specifically about the innovation |
|  | B3 | | Complexity | Perceived difficulty of implementation, reflected by duration, scope, radicalness, disruptiveness, centrality, and intricacy and number of steps required to implement.  🡪 If a statement regards user-friendliness, only code under II.B4. |
|  | B4 | | Design Quality and Packaging | Perceived excellence in how the intervention is bundled, presented, and assembled.  🡪 Also, code statements related to user-friendliness. |
|  | B5 | | Cost | Costs of the intervention and costs associated with implementing the intervention including investment, supply, and opportunity costs. |
|  | B6 | | Medical result [ADDED] | The relative advantage from medical/patient viewpoint. |
| **III. OUTER SETTING** | | | | |
| A | Patient Needs and Resources | | | The extent to which patient needs, as well as barriers and facilitators to meet those needs, are accurately known and prioritized by the organization.  🡪 Do not double-code possible advantages that can be mentioned to patients under II(.B6).  🡪 Patient needs must be specific (e.g., do not code “it has to benefit the patient” here. |
| B | Cosmopolitanism  Peer Pressure  Engaging: External Change Agents [PROCESS] | | | The degree to which an organization is networked with other external organizations.  Mimetic or competitive pressure to implement an intervention; typically because most or other key peer or competing organizations have already implemented or are in a bid for a competitive edge.  Individuals who are affiliated with an outside entity who formally influence or facilitate intervention decisions in a desirable direction.  🡪 Influence from other organizations. |
| C | External Policy and Incentives  Awareness [ADDED] | | | A broad construct that includes external strategies to spread interventions, including policy and regulations (governmental or other central entity), external mandates, recommendations and guidelines, pay-for-performance, collaboratives, and public or benchmark reporting.  The need for or lack of awareness among e.g., physicians, the general public.  🡪 Only code denying answers at this code. (“I can’t think of anything”). Other answers should be coded at one of the sub-codes. |
|  | C1 | Policy and legislation [ADDED] | | 🡪 Related to policy or legislation. |
|  | C2 | Incentives, awareness, structure [ADDED] | | 🡪 Other. E.g., use of AB and AMR in the Netherlands. |
| **IV. INNER SETTING** | | | | |
| A | Structural Characteristics | | | The social architecture, age, maturity, and size of an organization.  🡪 Including financing structure.  🡪 If a comment relates to implementation, code at IV.B. |
| B | Implementation Climate  Implementation Climate: Organizational Incentives & Rewards  Implementation Climate: Goals and Feedback  Implementation Climate: Learning Climate  Culture | | | The absorptive capacity for change, shared receptivity of involved individuals to an intervention, and the extent to which use of that intervention will be rewarded, supported, and expected within their organization.  Extrinsic incentives such as goal-sharing awards, performance reviews, promotions, and raises in salary, and less tangible incentives such as increased stature or respect.  The degree to which goals are clearly communicated, acted upon, and fed back to staff, and alignment of that feedback with goals.  A climate in which: a) leaders express their own fallibility and need for team members’ assistance and input; b) team members feel that they are essential, valued, and knowledgeable partners in the change process; c) individuals feel psychologically safe to try new methods; and d) there is sufficient time and space for reflective thinking and evaluation.  Norms, values, and basic assumptions of a given organization.  🡪 E.g., concurrent activities or priorities, possibilities for or experience with courses, whether costs can easily be covered. |
|  | B1 | Relevant stakeholders [ADDED]  Engaging [PROCESS]  Engaging: Opinion Leaders  Engaging: Formally Appointed Internal Implementation Leaders  Engaging: Champions  Readiness for Implementation: Leadership Engagement | | Individuals that might have a role in or an influence on adoption.  Attracting and involving appropriate individuals in the implementation and use of the intervention through a combined strategy of social marketing, education, role modeling, training, and other similar activities.  Individuals in an organization who have formal or informal influence on the attitudes and beliefs of their colleagues with respect to implementing the intervention.  Individuals from within the organization who have been formally appointed with responsibility for implementing an intervention as coordinator, project manager, team leader, or other similar role.  “Individuals who dedicate themselves to supporting, marketing, and ‘driving through’ an [implementation]” [101] (p. 182), overcoming indifference or resistance that the intervention may provoke in an organization.  Commitment, involvement, and accountability of leaders and managers with the implementation.  🡪 Only if specific individuals are mentioned. Information about the organization is coded under III.B. |
|  | B2 | Implementation Climate: Relative priority  Implementation Climate: Tension for Change | | Individuals’ shared perception of the importance of the implementation within the organization.  The degree to which stakeholders perceive the current situation as intolerable or needing change.  🡪 Answer to “Is it needed, is it an addition or replacement, are there any alternatives you prefer?” |
|  | B3 | Implementation Climate: Compatibility  Readiness for Implementation: Available Resources | | The degree of tangible fit between meaning and values attached to the intervention by involved individuals, how those align with individuals’ own norms, values, and perceived risks and needs, and how the intervention fits with existing workflows and systems.  The level of resources dedicated for implementation and on-going operations, including money, training, education, physical space, and time.  🡪 Only code practical characteristics here. Other related characteristics can be coded at IV.B. |

Most constructs and definitions were adopted from the Consolidated Framework for Implementation Research (CFIR; Damschroder et al., 2009). If codes were added or moved to a different topic (i.e., individual characteristics, outer setting, inner setting, innovation characteristics, process), this is indicated).

The following CFIR constructs were excluded from the coding tree. Process: Planning, Reflecting & Evaluating. Inner setting: Readiness for Implementation (i.e., Access to Knowledge and Information). Individual characteristics: Individual Identification with Organization. NB. The subconstructs belonging to the main construct ‘process’ are included under other constructs.

AB: antibiotics; AMR: antimicrobial resistance.
